# Real-world evidence from Japan regarding survival outcomes and treatment sequence in patients receiving CDK4/6 inhibitor plus endocrine therapy as first- or second-line treatment for hormone receptor–positive, HER2-negative advanced or metastatic breast cancer

**DOI:** 10.1007/s12282-025-01713-7

**Published:** 2025-05-20

**Authors:** Tetsuhiro Yoshinami, Yuko Takano, Yukinori Ozaki, Yukiko Kajiwara, Mitsugu Yamamoto, Ken-ichi Watanabe, Masami Tsukabe, Fumie Fujisawa, Shigenori E. Nagai, Nobuhiro Shibata, Chiya Oshiro, Hiroko Bando, Nobuyuki Tsunoda, Kazuhiko Yamagami, Kei Koizumi, Masahiro Takada, Naoko Toriguchi, Nobuyuki Sekine, Tsutomu Kawaguchi, Shigehira Saji, Yasuaki Sagara, Satoshi Morita, Norikazu Masuda

**Affiliations:** 1 Department of Breast and Endocrine Surgery, Graduate School of Medicine, Osaka University, 2-2-E10 Yamadaoka, Suita, Osaka 565-0871 Japan; 2 Department of Breast and Endocrine Surgery, Nagoya University Hospital, Nagoya, Aichi Japan; 3 Department of Breast Medical Oncology, The Cancer Institute Hospital of JFCR, Tokyo, Japan; 4 Department of Breast Surgery, Hiroshima City Hiroshima Citizens Hospital, Hiroshima, Japan; 5 Department of Breast Oncology, National Hospital Organization Hokkaido Cancer Center, Sapporo, Hokkaido Japan; 6 Department of Medical Oncology, Shiga General Hospital, Moriyama, Shiga Japan; 7 Division of Breast Oncology, Saitama Cancer Center, Kitaadachi-gun, Saitama, Japan; 8 Department of Clinical Oncology, Kansai Medical University Hospital, Hirakata, Osaka Japan; 9 Department of Breast Surgery, Kaizuka City Hospital, Kaizuka, Osaka Japan; 10 Department of Breast and Endocrine Surgery, Institute of Medicine, University of Tsukuba, Tsukuba, Ibaraki Japan; 11 Department of Breast Surgery, Japanese Red Cross Aichi Medical Center Nagoya Daiichi Hospital, Nagoya, Aichi Japan; 12 Department of Breast Surgery and Oncology, Shinko Hospital, Kobe, Hyogo Japan; 13 Department of Surgery 1, Division of Breast Surgery, Hamamatsu University School of Medicine, Hamamatsu, Shizuoka Japan; 14 Department of Breast Surgery, Kansai Medical University, Hirakata, Osaka Japan; 15 Department of Japan Drug Development and Medical Affairs, Eli Lilly Japan K.K, Kobe, Hyogo Japan; 16 Department of Medical Oncology, Fukushima Medical University, Fukushima, Japan; 17 Department of Breast and Thyroid Surgical Oncology, Social Medical Corporation Hakuaikai Sagara Hospital, Kagoshima, Japan; 18 Department of Biomedical Statistics and Bioinformatics, Graduate School of Medicine, Kyoto University, Kyoto, Japan; 19 Department of Breast Surgery, Graduate School of Medicine, Kyoto University, Kyoto, Japan

**Keywords:** Abemaciclib, Advanced or metastatic breast cancer, Cyclin-dependent kinase 4/6 inhibitor, Palbociclib, Real-world evidence

## Abstract

**Background:**

A cyclin-dependent kinase 4/6 inhibitor (CDK4/6i) plus endocrine therapy (ET) is a current standard first-/second-line treatment for hormone receptor (HR)-positive, HER2-negative advanced/metastatic breast cancer (AMBC). We aimed to provide real-world evidence regarding CDK4/6i therapy in this population.

**Methods:**

In this multicenter observational study, data from patients who had started CDK4/6i therapy between January 1, 2019, and December 31, 2021, as first-/second-line treatment for AMBC were used; real-world progression-free survival (rwPFS), chemotherapy-free survival, and overall survival were analyzed using the Kaplan–Meier method. Additionally, data were analyzed by separating patients with treatment-free interval (TFI) < 12 months (deemed resistant to ET) from the first-line treatment group (hereafter, the exclusive first-line treatment group).

**Results:**

Data from 745 patients were analyzed. Compared with palbociclib, abemaciclib was used in younger patients and those with expected poor prognosis. Median rwPFS was 36.8, 17.8, and 31.4 months in patients with de novo stage IV disease, TFI < 12 months, and TFI ≥ 12 months, respectively, in the first-line treatment group, and 17.4 months in the second-line treatment group. In the exclusive first-line treatment group, median rwPFS of the subsequent treatment after initial CDK4/6i plus ET was < 7 months, regardless of the type of subsequent treatment; prognosis was especially poor in those who were switched to chemotherapy.

**Conclusions:**

The real-world survival outcomes found in this study for patients receiving first-/second-line CDK4/6i therapy were consistent with those of randomized phase 3 studies. As outcomes of subsequent treatment after initial CDK4/6i plus ET remain insufficient, further improvement in treatment is necessary.

**Supplementary Information:**

The online version contains supplementary material available at 10.1007/s12282-025-01713-7.

## Introduction

Hormone receptor (HR)-positive, human epidermal growth factor receptor 2 (HER2)-negative breast cancer is the most common subtype of breast cancer [1]. The combination of a cyclin-dependent kinase 4/6 inhibitor (CDK4/6i) and endocrine therapy (ET) is a current standard first- and second-line treatment for advanced or metastatic breast cancer (AMBC) in this population [2, 3]. The choice of CDK4/6i for first- or later-line treatment is largely left to the discretion of individual physicians based on the needs of each patient.

In Japan, the CDK4/6is that have been approved to date and recommended by guidelines [4] are palbociclib and abemaciclib. Each drug prolongs progression-free survival (PFS) in patients with AMBC when combined with fulvestrant as first- or second-line treatment [5–8] or with a non-steroidal aromatase inhibitor (AI) as first-line treatment [9–11]; however, in terms of overall survival (OS), evidence for benefits of adding CDK4/6i to ET has been less clear [12]. In the first-line setting, abemaciclib combined with an AI (anastrozole or letrozole) has produced a clinically meaningful (but not statistically significant) improvement in OS [13, 14]; however, no clear evidence of improvement was found with palbociclib plus letrozole [15].

Moreover, in cases of disease progression despite treatment with a CDK4/6i plus ET, optimal subsequent treatments are yet to be established. Post-CDK4/6i treatment options include ET alone, ET plus a CDK4/6i or a molecularly-targeted agent (e.g. everolimus), or chemotherapy. Regarding the option of CDK4/6i with ET, the findings to date are inconsistent; although addition of ribociclib (as in the phase 2 MAINTAIN trial) [16] or abemaciclib (as in the phase 3 postMONARCH trial) [17] to ET produced a significant improvement in PFS when compared with ET alone, no such improvement was observed with palbociclib [18]. Currently, no established guideline based on randomized controlled studies (RCTs) exists regarding next-line systemic treatment after progression on a CDK4/6i plus ET [19].

In the present study, we aimed to clarify characteristics and survival outcomes of patients who had received a CDK4/6i (palbociclib or abemaciclib) in combination with ET as first- or second-line treatment for HR-positive, HER2-negative AMBC in daily clinical practice in Japan. In addition, we investigated what post-CDK4/6i treatments were being used following progression on a CDK4/6i plus ET, as well as their outcomes, in order to address a knowledge gap in the current evidence. For this purpose, we conducted exploratory analyses for real-world PFS (rwPFS) and OS in patients who had early disease progression and received subsequent treatment after first- or second-line CDK4/6i plus ET.

## Patients and methods

### Study design

This is a multicenter observational study designed to obtain and analyze real-world data on the use of a CDK4/6i in combination with ET as first- or second-line treatment for HR-positive, HER2-negative AMBC. Anonymized data were collected, via an electronic data capture system, from all patients who had started treatment with a CDK4/6i (palbociclib or abemaciclib) from January 1, 2019, to December 31, 2021, at 40 participating institutions with breast cancer or medical oncology specialists across Japan. We attempted to minimize bias by ensuring inclusion of consecutive patients during the prespecified period, and the study was carried out by highly experienced physicians at specialist sites. Data collected to June 30, 2023, were used for the analysis described in the present article. The study is registered with the University Hospital Medical Information Network Clinical Trials Registry (UMIN000051975).

### Data source and patients

Relevant data from existing data sources, including electronic medical records, were collected for women, regardless of menopausal status, with a diagnosis of AMBC (i.e. breast cancer with unresectable local disease or distant metastases) and who met the following criteria: age, ≥ 18 years when informed consent was obtained or, in cases of opt-out consent, at the time of data entry; estrogen receptor–positive or progesterone receptor–positive status confirmed by immunohistochemistry (IHC) or an Allred proportion score of ≥ 2; and HER2-negative status confirmed by IHC score 0 or 1 +, or negative results on fluorescence or dual in situ hybridization.

Patients were eligible for inclusion if, based on drug-prescription data at the participating study site, they had between January 1, 2019, and December 31, 2021, received a CDK4/6i (palbociclib or abemaciclib) in combination with ET as first- or second-line treatment for AMBC. In this context, first-line treatment was defined as the first treatment after diagnosis of AMBC, and second-line treatment as the one immediately after that. In cases in which patients started treatment with ET alone and a CDK4/6i was added before disease progression, it was considered a single line of therapy.

In all cases, patients had received no chemotherapy before the start of CDK4/6i therapy as treatment for AMBC, and palbociclib or abemaciclib had been administered for ≥ 2 months. In cases in which palbociclib or abemaciclib was used perioperatively, a period of ≥ 6 months was required between the diagnosis of AMBC and the last administration of the drug.

### Analyses of survival outcomes

The following survival outcomes were evaluated: rwPFS, chemotherapy-free survival (CFS), and OS. rwPFS was defined as the time from the start of the relevant treatment for AMBC to death from any cause or disease progression (based on data collected from the medical records of each patient), whichever occurs earlier. CFS was defined as the time from the start of the relevant treatment for AMBC to the start of chemotherapy or death from any cause, whichever occurs earlier. Chemotherapy was defined as treatment with any cytotoxic anticancer agent administered by any route (including oral administration). OS was defined as the time from the start of the relevant treatment for AMBC to death from any cause.

### Classification of patients for analyses

As per the study protocol, data were primarily analyzed by treatment line. For brevity, patients receiving a CDK4/6i as first-line or second-line treatment for AMBC are referred to as the ‘first-line treatment group’ and the ‘second-line treatment group’, respectively.

In the first-line treatment group, patients were divided into three groups according to the following criteria: de novo stage IV disease, and treatment-free interval (TFI) (defined as the time from the end of adjuvant ET to diagnosis of AMBC) < 12 months and TFI ≥ 12 months.

Because patients with TFI < 12 months are deemed to have low sensitivity to ET [20], subsequent analyses were carried out by dividing patients into the following groups.

- Exclusive first-line treatment group: patients with de novo stage IV disease or TFI ≥ 12 months in the first-line treatment group.
- Expanded second-line treatment group: patients with TFI < 12 months in the first-line treatment group and those in the second-line treatment group.

Additionally, in patients who required further treatment after having disease progression while receiving a CDK4/6i plus ET, survival outcomes were analyzed by type of subsequent treatment that was given immediately after the initial CDK4/6i plus ET: ET, molecularly-targeted therapy (excluding CDK4/6is), continuous CDK4/6i therapy, or chemotherapy.

### Statistical analyses

The sample size was determined as follows. Based on the results of previous studies [10, 11, 16, 21], the threshold and expected values of CFS were estimated to be 30 months and 45 months, respectively. With the alpha error set at 5%, 122 cases were required for analysis per group. Considering that the data were to be analyzed by several subgroups, a minimum sample size of 400 cases was needed.

Analyses were carried out using data from the full analysis set (FAS), subdivided into groups as described above. To assess intergroup differences in patient characteristics by type of CDK4/6i used (palbociclib or abemaciclib), which is one of the aims of the present study, the Wilcoxon rank-sum test was used for continuous variables, Fisher’s exact test for dichromatic variables, and the chi-squared test for categorical variables. For the analyses of rwPFS, CFS, and OS, the Kaplan–Meier method was used to generate survival curves, and the log-rank test was used for comparisons. Statistical analyses were carried out using R 4.4.1 (R Foundation for Statistical Computing, Vienna, Austria).

## Results

### Patient characteristics

**Fig. 1:**
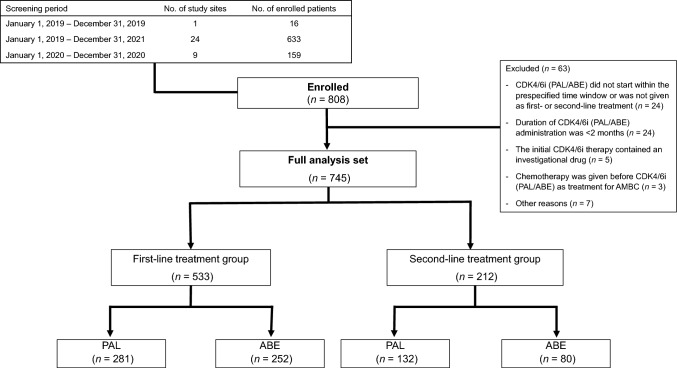
Patient disposition. *ABE* abemaciclib; *CDK4/6i* cyclin-dependent kinase 4/6 inhibitor; *PAL* palbociclib

A total of 808 patients with AMBC were registered. After exclusion of 63 patients, the FAS comprised data from 745 patients, of whom 533 and 212 received CDK4/6i therapy as first-line treatment and second-line treatment for AMBC, respectively (Fig. 1). Of the 212 patients who received CDK4/6i therapy as second-line treatment, 112 (52.8%) had been treated with an AI ± luteinizing hormone releasing hormone analog (LHRHa), 53 (25.0%) with fulvestrant ± LHRHa, 45 (21.2%) with a selective estrogen receptor modulator ± LHRHa, and 2 (0.9%) with other treatments. The median follow-up was 32.5 (range 2.5–101.0) months.

Table 1 summarizes the characteristics of the patients in each of the two treatment-line groups, subdivided by type of CDK4/6i received (palbociclib or abemaciclib). In the first-line treatment group, patients who received abemaciclib were generally younger than those who received palbociclib (median age, 57.0 vs 62.0 years; *p* < 0.001). They were also more likely than patients in the palbociclib group to have TFI < 12 months (78.5% vs 60.1%; *p* < 0.001) and Ki67 ≥ 30% (48.2% vs 35.5%; *p* = 0.013); their non-luminal disease score (NOLUS) [22] was generally higher (median, 33.50 vs 28.54; *p* = 0.026); and they were more likely to have liver metastasis (32.9% vs 13.5%; *p* = < 0.001) and have received perioperative chemotherapy (50.4% vs 39.1%; *p* = 0.012). In the second-line treatment group, no differences were found between patients who received abemaciclib and those who received palbociclib, except that the former were generally younger (median age, 57.0 vs 60.0 years; *p* = 0.008) and less likely to be postmenopausal (63.8% vs 75.8%; *p* = 0.035). Regarding employment status, no differences were found in either the first- or the second-line treatment group between patients who received palbociclib and those who received abemaciclib.

**Table 1:** Patient characteristics

	Full analysis set (*n* = 745)	First-line treatment group (*n* = 533)			Second-line treatment group (*n* = 212)		
		Palbociclib (*n* = 281)	Abemaciclib (*n* = 252)	*p*-value	Palbociclib (*n* = 132)	Abemaciclib (*n* = 80)	*p*-value
Patient characteristics							
Age, years							
Median (range)	60.0 (31–93)	62.0 (32–88)	57.0 (31–80)	< 0.001*	60.0 (37–93)	57.0 (37–83)	0.008*
< 75 years	661 (88.7)	239 (85.1)	236 (93.7)	0.002**	111 (84.1)	75 (93.8)	0.051**
≥ 75 years	84 (11.3)	42 (14.9)	16 (6.3)		21 (15.9)	5 (6.2)	
AMBC type							
Distant recurrence after curative surgery	503 (67.5)	186 (66.2)	174 (69.0)	0.524***	88 (66.7)	55 (68.8)	0.704***
De novo stage IV	230 (30.9)	90 (32.0)	76 (30.2)		40 (30.3)	24 (30.0)	
Curatively unresectable local disease (without distant metastases)	12 (1.6)	5 (1.8)	2 (0.8)		4 (3.0)	1 (1.2)	
Treatment-free interval							
< 12 months	299 (65.9^a^)	104 (60.1^a^)	124 (78.5^a^)	< 0.001** (excluding NE cases)	42 (56.8^a^)	29 (59.2^a^)	0.853** (excluding NE cases)
≥ 12 months	155 (34.1^a^)	69 (39.9^a^)	34 (21.5^a^)		32 (43.2^a^)	20 (40.8^a^)	
NE (date of last dose of adjuvant ET is unknown)	49	13	16		14	6	
HR status							
ER-positive	742 (99.7^a^)	280 (99.6^a^)	251 (99.6^a^)	1.000** (excluding NE cases)	132 (100.0^a^)	79 (100.0^a^)	NA
ER-negative	2 (0.3^a^)	1 (0.4^a^)	1 (0.4^a^)		0 (0^a^)	0 (0^a^)	
Unknown (NE)	1	0	0		0	1	
ER-positive cells ≥ 10%	472 (99.4^a^)	188 (98.9^a^)	153 (99.4^a^)	0.692* (excluding NE cases)	79 (100.0^a^)	52 (100.0^a^)	NA
ER-positive cells < 10%	3 (0.6^a^)	2 (1.1^a^)	1 (0.6^a^)		0 (0^a^)	0 (0^a^)	
Unknown (NE)	270	91	98		53	28	
PgR-positive	623 (83.7^a^)	237 (84.3^a^)	211 (83.7^a^)	0.906** (excluding NE cases)	106 (80.3^a^)	69 (87.3^a^)	0.256** (excluding NE cases)
PgR-negative	121 (16.3^a^)	44 (15.7^a^)	41 (16.3^a^)		26 (19.7^a^)	10 (12.7^a^)	
Unknown (NE)	1	0	0		0	1	
PgR-positive cells ≥ 10%	343 (73.6^a^)	136 (72.7^a^)	115 (77.7^a^)	0.345* (excluding NE cases)	52 (66.7^a^)	40 (75.5^a^)	0.173* (excluding NE cases)
PgR-positive cells < 10%	123 (26.4^a^)	51 (27.3^a^)	33 (22.3^a^)		26 (33.3^a^)	13 (24.5^a^)	
Unknown (NE)	279	94	104		54	27	
HER2 status							
Positive	0 (0.0)	0 (0)	0 (0)	NA	0 (0.0)	0 (0)	NA
Negative	744 (99.9)	281 (100.0)	252 (100.0)		132 (100.0)	79 (98.8)	
Unknown (NE)	1 (0.1)	0 (0)	0 (0)		0 (0.0)	1 (1.2)	
Histological grade							
Grade 1	49 (15.3^a^)	21 (16.8^a^)	15 (12.9^a^)	0.088* (excluding NE cases)	5 (11.1^a^)	8 (23.5^a^)	0.832* (excluding NE cases)
Grade 2	207 (64.7^a^)	85 (68.0^a^)	73 (62.9^a^)		32 (71.1^a^)	17 (50.0^a^)	
Grade 3	64 (20.0^a^)	19 (15.2^a^)	28 (24.1^a^)		8 (17.8^a^)	9 (26.5^a^)	
Unknown (NE)	425	156	136		87	46	
Ki67							
< 10%	87 (15.8^a^)	39 (17.7^a^)	26 (13.6^a^)	0.013* (excluding NE cases)	13 (14.1^a^)	9 (18.4^a^)	0.659* (excluding NE cases)
≥ 10%, < 30%	242 (43.8^a^)	103 (46.8^a^)	73 (38.2^a^)		47 (51.1^a^)	19 (38.8^a^)	
≥ 30%	223 (40.4^a^)	78 (35.5^a^)	92 (48.2^a^)		32 (34.8^a^)	21 (42.9^a^)	
NE	193	61	61		40	31	
NOLUS^b^							
Median (range)	29.63 (1.1–78.8)	28.54 (1.1–78.8)	33.50 (2.7–66.7)	0.026*	28.98 (4.0–69.4)	25.40 (2.7–77.9)	0.762*
Negative < 51.38	260 (90.9^a^)	113 (92.6^a^)	87 (88.8^a^)	0.353** (excluding NE cases)	42 (93.3^a^)	18 (85.7^a^)	0.373** (excluding NE cases)
Positive ≥ 51.38	26 (9.1^a^)	9 (7.4^a^)	11 (11.2^a^)		3 (6.7^a^)	3 (14.3^a^)	
Unknown (NE)	459	159	154		87	59	
Perioperative treatment							
Time between date of surgery and date of AMBC diagnosis, years							
Median (range)	5.3 (0.1–34.3)	6.0 (0.5–33.4)	4.2 (0.3–34.3)	< 0.001*	6.0 (1.0–31.5)	5.3 (0.1–24.3)	0.681*
Unknown (NE)	1	1	0		0	0	
Perioperative therapy							
No	26 (5.1)	10 (5.3)	5 (2.9)	0.355***	6 (6.7)	5 (8.9)	0.856***
Yes	482 (94.9)	177 (94.7)	170 (97.1)		84 (93.3)	51 (91.1)	
ET alone^c^	434 (58.3)	159 (56.6)	157 (62.3)	0.210***	70 (53.0)	48 (60.0)	0.397***
Chemotherapy ± other therapy^c^	321 (43.1)	110 (39.1)	127 (50.4)	0.012***	48 (36.4)	36 (45.0)	0.271***
Molecular targeted therapy ± ET^c^ (excluding chemotherapy)	11 (1.5)	3 (1.1)	5 (2.0)	0.609***	1 (0.8)	2 (2.5)	0.659***
Status at start of the first-line treatment for AMBC							
PS^d^							
0	496 (66.8^a^)	182 (64.8^a^)	174 (69.0^a^)	0.412* (excluding NE cases)	81 (62.8^a^)	59 (73.8^a^)	0.091* (excluding NE cases)
1	162 (21.8^a^)	70 (24.9^a^)	49 (19.4^a^)		29 (22.5^a^)	14 (17.5^a^)	
≥ 2	84 (11.3^a^)	29 (10.3^a^)	29 (11.5^a^)		19 (14.7^a^)	7 (8.8^a^)	
Unknown (NE)	3	0	0		3	0	
Menopausal status							
Premenopausal	180 (24.8^a^)	49 (17.9^a^)	76 (30.8^a^)	< 0.001** (excluding NE cases)	27 (21.3^a^)	28 (35.4^a^)	0.035** (excluding NE cases)
Postmenopausal	547 (75.2^a^)	225 (82.1^a^)	171 (69.2^a^)		100 (78.7^a^)	51 (64.6^a^)	
Unknown (NE)	18	7	5		5	1	
Employment status at start of first-line treatment for AMBC							
No	269 (60.4^a^)	107 (61.5^a^)	94 (58.4^a^)	0.578** (excluding NE cases)	42 (65.6^a^)	26 (56.5^a^)	0.426** (excluding NE cases)
Yes	176 (39.6^a^)	67 (38.5^a^)	67 (41.6^a^)		22 (34.4^a^)	20 (43.5^a^)	
Unknown (NE)	300	107	91		68	34	
Metastasis							
No	1 (0.1)	0 (0)	1 (0.4)	0.473**	0 (0)	0 (0)	NA
Yes	744 (99.9)	281 (100.0)	251 (99.6)		132 (100.0)	80 (100)	
Bone^c^	436 (58.5)	169 (60.1)	144 (57.1)	0.537**	77 (58.3)	46 (57.5)	1.000**
Lung^c^	225 (30.2)	86 (30.6)	84 (33.3)	0.516**	38 (28.8)	17 (21.2)	0.260**
Distant lymph nodes^c^	203 (27.2)	70 (24.9)	77 (30.6)	0.147**	37 (28.0)	19 (23.8)	0.524**
Liver^c^	146 (19.6)	38 (13.5)	83 (32.9)	< 0.001**	14 (10.6)	11 (13.8)	0.515**
Pleura^c^	88 (11.8)	36 (12.8)	28 (11.1)	0.595**	15 (11.4)	9 (11.2)	1.000**
Central nervous system (including cancerous meningitis)^c^	18 (2.4)	7 (2.5)	5 (2.0)	0.776**	2 (1.5)	4 (5.0)	0.202**

Data are expressed as n (%) unless otherwise specified

*AMBC* advanced or metastatic breast cancer; *ER* endocrine receptor; *ET* endocrine therapy; *HER2* human epidermal growth factor receptor 2; *HR* hormone receptor; *NA* not applicable; *NE* not evaluable; *NOLUS* non-luminal disease score; *PgR* progesterone receptor; *PS* performance status; *SD* standard deviation

^*^Wilcoxon rank-sum test

^**^Fisher’s exact test

^***^chi-squared test

^a^The percentages were calculated using numbers excluding missing (unknown) values as a denominator. A significance test was conducted using these values

^b^NOLUS was calculated using the formula: − 0.45 × ER (%) − 0.28 × PgR (%) + 0.27 × Ki67 (%) + 73. ER (%), PgR (%) and Ki67 (%) indicate the actual percentage of respective cells. In case any of the percentages is missing, NOLUS is deemed not evaluable

^c^The count may be duplicated

^d^PS in the medical record was used when available. For patients whose PS was not specified in the medical record (579/745; 77.7%), PS was determined as follows: without cancer symptoms or adverse effect (PS 0), with cancer symptoms or adverse effect but no limitation of daily activities (PS 1), with limitation of daily activities (PS ≥ 2), or with activities of daily living dependency (PS ≥ 2)

### Survival outcomes by treatment line

**Fig. 2:**
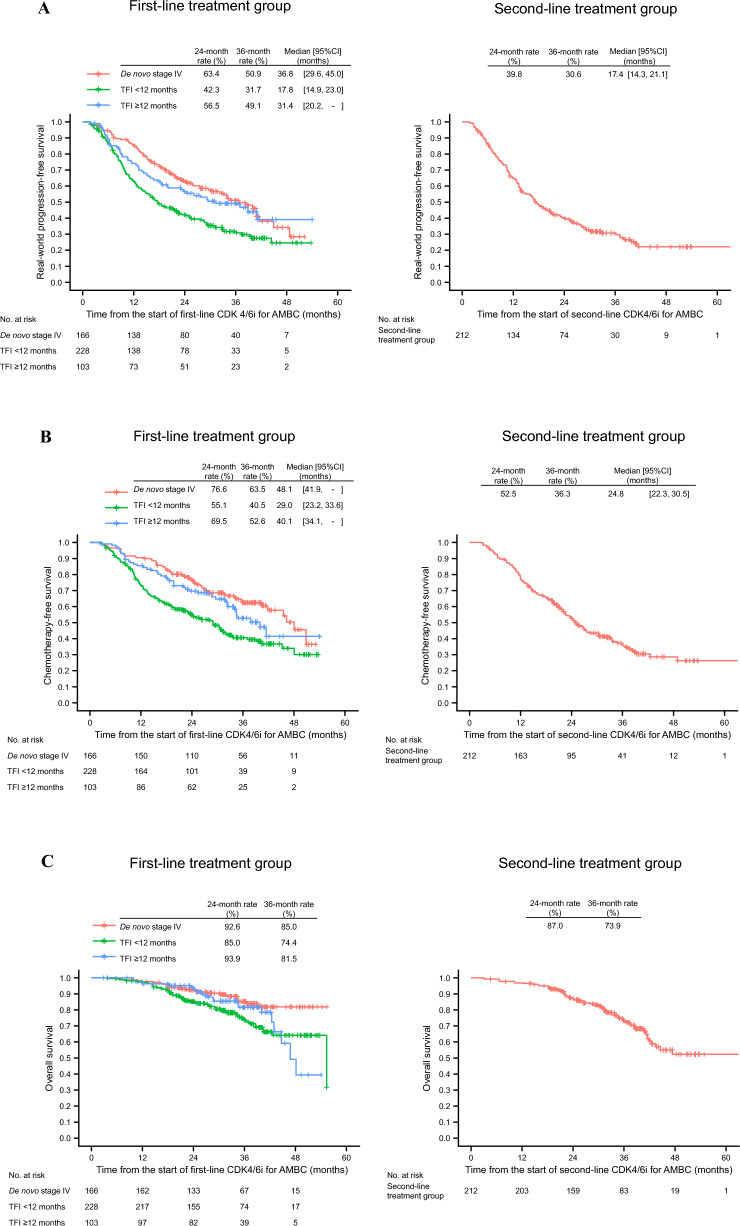
Survival outcomes in patients in the first- and second-line treatment groups: real-world progression-free survival (**A**), chemotherapy-free survival (**B**), and overall survival (**C**). *AMBC* advanced or metastatic breast cancer; *CDK4/6i* cyclin-dependent kinase 4/6 inhibitor; *CI* confidence interval; *TFI* treatment-free interval

Figure 2A shows the results for rwPFS. In the first-line treatment group, median rwPFS (95% CI) was 36.8 (29.6–45.0), 17.8 (14.9–23.0), and 31.4 (20.2 to not reached) months in patients with de novo stage IV disease, TFI < 12 months, and TFI ≥ 12 months, respectively. Median rwPFS in patients with TFI < 12 months was markedly shorter than that in either of the other two groups (de novo stage IV disease [reference] vs TFI < 12 months, log-rank *p* < 0.001; TFI < 12 months [reference] vs TFI ≥ 12 months, log-rank *p* = 0.009). In the second-line treatment group, median rwPFS (defined as time from the start of second-line CDK4/6i for AMBC) (95% CI) was 17.4 (14.3–21.1) months.

Figure 2B shows the results for CFS. In the first-line treatment group, median CFS (95% CI) was 48.1 (41.9 to not reached), 29.0 (23.2–33.6), and 40.1 (34.1 to not reached) months in patients with de novo stage IV disease, TFI < 12 months, and TFI ≥ 12 months, respectively. Median CFS in patients with TFI < 12 months was markedly shorter than that in either of the other two groups (de novo stage IV disease [reference] vs TFI < 12 months, log-rank *p* < 0.001; TFI < 12 months [reference] vs TFI ≥ 12 months, log-rank *p* = 0.013). In the second-line treatment group, median CFS (defined as time from the start of the second-line treatment) (95% CI) was 24.8 (22.3–30.5) months.

Figure 2C shows the results for OS. In the first-line treatment group, the 2-year OS rate was 92.6%, 85.0%, and 93.9%, and the 3-year OS rate was 85.0%, 74.4%, and 81.5%, in patients with de novo stage IV disease, TFI < 12 months, and TFI ≥ 12 months, respectively. OS in patients with TFI < 12 months was markedly shorter than that in patients with de novo stage IV disease (de novo stage IV disease [reference] vs TFI < 12 months, HR 2.049 [95% CI, 1.251–3.357], log-rank *p* = 0.004). In the second-line treatment group, the 3-year OS rate was 73.9%.

Survival outcomes by CDK4/6i in the exclusive first-line treatment group appear to show a similar tendency; with palbociclib and abemaciclib, respectively, the 3-year rwPFS rate was 47.6% and 54.2% (Supplementary Fig. 1 A, B), the 3-year CFS rate was 58.8% and 60.4% (Supplementary Fig. 2 A, B), and the 3-year OS rate was 85.8% and 80.6% (Supplementary Fig. 3 A, B).

### Type of endocrine therapy used with CDK4/6i

We analyzed rwPFS by type of ET (i.e. fulvestrant or AI) used with CDK4/6i in the exclusive first-line treatment group in order to investigate possible differences associated with the use of concomitant endocrine agents in endocrine-sensitive patients; no differences in rwPFS were found between the two groups (Supplementary Fig. 4).

### Type and outcomes of subsequent treatment after CDK4/6i plus ET

Of the total 745 patients enrolled, 334 patients are continuing to receive the initial CDK4/6i therapy plus ET, whereas treatment was switched for the 411 patients who had disease progression. Table 2 summarize the subsequent treatments; 167 (40.6%) received chemotherapy; oral fluoropyrimidine (S-1 and capecitabine)-containing regimens were most frequently used (86/167), whereas taxane (paclitaxel)-containing therapy was used in relatively few patients (50/167, including 41 who received paclitaxel plus bevacizumab).

**Table 2:** Subsequent treatment after initial cyclin-dependent kinase 4/6 inhibitor (CDK4/6i) plus endocrine therapy (ET)

	*n* (%)^a^	Exclusive first-line treatment group			Expanded second-line treatment group		
		Palbociclib	Abemaciclib	Total	Palbociclib	Abemaciclib	Total
Total	411 (100.0)	78 (100.0)	49 (100.0)	127 (100.0)	154 (100.0)	130 (100.0)	284 (100.0)
Palbociclib (PAL) + ET	22 (5.4)	4 (5.1)	6 (12.2)	10 (7.9)	6 (3.9)	6 (4.6)	12 (4.2)
PAL + fulvestrant	17 (4.1)	4 (5.1)	6 (12.2)	10 (7.9)	5 (3.2)	2 (1.5)	7 (2.5)
PAL + other	5 (1.2)	–	–	–	1 (0.6)	4 (3.1)	5 (1.8)
Abemaciclib (ABE) + ET	58 (14.1)	23 (29.5)	4 (8.2)	27 (21.3)	25 (16.2)	6 (4.6)	31 (10.9)
ABE + fulvestrant	37 (9.0)	14 (17.9)	3 (6.1)	17 (13.4)	16 (10.4)	4 (3.1)	20 (7.0)
ABE + letrozole	6 (1.5)	3 (3.8)	–	3 (2.4)	2 (1.3)	1 (0.8)	3 (1.1)
ABE + anastrozole	5 (1.2)	3 (3.8)	–	3 (2.4)	1 (0.6)	1 (0.8)	2 (0.7)
ABE + exemestane	3 (0.7)	–	–	–	3 (1.9)	–	3 (1.1)
ABE + fulvestrant/LHRH analog	3 (0.7)	1 (1.3)	–	1 (0.8)	2 (1.3)	–	2 (0.7)
ABE + other	4 (1.0)	2 (2.6)	1 (2.0)	3 (2.4)	1 (0.6)	–	1 (0.4)
ET alone	70 (17.0)	20 (25.6)	12 (24.5)	32 (25.2)	19 (12.3)	19 (14.6)	38 (13.4)
Fulvestrant	31 (7.5)	16 (20.5)	9 (18.4)	25 (19.7)	2 (1.3)	4 (3.1)	6 (2.1)
Toremifene	11 (2.7)	–	–	–	7 (4.5)	4 (3.1)	11 (3.9)
Letrozole	7 (1.7)	2 (2.6)	2 (4.1)	4 (3.1)	2 (1.3)	1 (0.8)	3 (1.1)
Letrozole/LHRH analog	5 (1.2)	–	–	–	2 (1.3)	3 (2.3)	5 (1.8)
Tamoxifen	5 (1.2)	1 (1.3)	–	1 (0.8)	1 (0.6)	3 (2.3)	4 (1.4)
Exemestane	4 (1.0)	–	–	–	2 (1.3)	2 (1.5)	4 (1.4)
Medroxyprogesterone acetate	4 (1.0)	1 (1.3)	–	1 (0.8)	2 (1.3)	1 (0.8)	3 (1.1)
Other	3 (0.7)	–	1 (2.0)	1 (0.8)	1 (0.6)	1 (0.8)	2 (0.7)
Molecular targeted therapy (excluding CDK4/6i) ± ET (without chemotherapy)	94 (22.9)	15 (19.2)	6 (12.2)	21 (16.5)	40 (26.0)	33 (25.4)	73 (25.7)
Everolimus + exemestane	71 (17.3)	11 (14.1)	6 (12.2)	17 (13.4)	36 (23.4)	18 (13.8)	54 (19.0)
Olaparib	10 (2.4)	–	–	–	2 (1.3)	8 (6.2)	10 (3.5)
IND (molecular-targeted agent) + fulvestrant	5 (1.2)	3 (3.8)	–	3 (2.4)	–	2 (1.5)	2 (0.7)
Everolimus + exemestane/LHRH analog	5 (1.2)	1 (1.3)	–	1 (0.8)	1 (0.6)	3 (2.3)	4 (1.4)
Other (including everolimus)	3 (0.7)	–	–	–	1 (0.6)	2 (1.5)	3 (1.1)
Chemotherapy ± other treatment	167 (40.6)	16 (20.5)	21 (42.9)	37 (29.1)	64 (41.6)	66 (50.8)	130 (45.8)
S-1	52 (12.7)	4 (5.1)	5 (10.2)	9 (7.1)	19 (12.3)	24 (18.5)	43 (15.1)
Paclitaxel + bevacizumab	41 (10.0)	2 (2.6)	8 (16.3)	10 (7.9)	13 (8.4)	18 (13.8)	31 (10.9)
Capecitabine	25 (6.1)	1 (1.3)	2 (4.1)	3 (2.4)	13 (8.4)	9 (6.9)	22 (7.7)
Eribulin	15 (3.6)	2 (2.6)	–	2 (1.6)	8 (5.2)	5 (3.8)	13 (4.6)
Capecitabine/cyclophosphamide	9 (2.2)	3 (3.8)	–	3 (2.4)	3 (1.9)	3 (2.3)	6 (2.1)
Paclitaxel	6 (1.5)	2 (2.6)	1 (2.0)	3 (2.4)	2 (1.3)	1 (0.8)	3 (1.1)
Epirubicin/cyclophosphamide	4 (1.0)	–	2 (4.1)	2 (1.6)	–	2 (1.5)	2 (0.7)
Epirubicin/cyclophosphamide/fluorouracil	4 (1.0)	1 (1.3)	1 (2.0)	2 (1.6)	1 (0.6)	1 (0.8)	2 (0.7)
Paclitaxel + bevacizumab/IND (immune checkpoint inhibitor)	3 (0.7)	–	2 (4.1)	2 (1.6)	–	1 (0.8)	1 (0.4)
Other	8 (1.9)	1 (1.3)	–	1 (0.8)	5 (3.2)	2 (1.5)	7 (2.5)

*IND* investigational drug; *LHRH* luteinizing hormone releasing hormone

^a^Number of patients who received subsequent treatment after the initial CDK4/6i therapy in the exclusive first-line treatment group and the expanded second-line treatment group

**Fig. 3:**
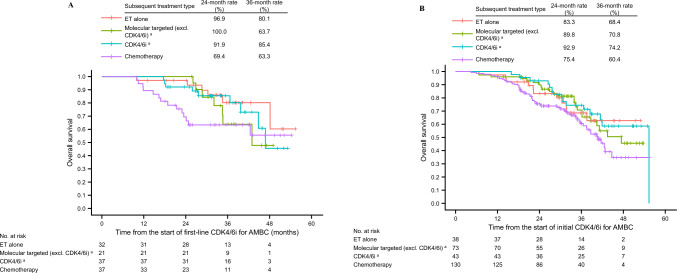
Overall survival by subsequent treatment after initial CDK4/6i plus ET in the exclusive first-line treatment group (**A**) and expanded second-line treatment group (**B**). *AMBC* advanced or metastatic breast cancer; *CDK4/6i* cyclin-dependent kinase 4/6 inhibitor; *CI* confidence interval; *ET* endocrine therapy. ^a^Treatment was provided with or without ET

In those patients who had disease progression on the initial CDK4/6i plus ET, we analyzed OS by subsequent treatment after initial CDK4/6i plus ET in order to investigate survival outcomes by type of post-CDK4/6i treatment. Figure 3A shows OS in the exclusive first-line treatment group (i.e. time from the start of first-line CDK4/6i for AMBC); the 2-year OS rate was 96.9%, 100%, 91.9%, and 69.4% in the ET alone, molecularly-targeted therapy (excluding CDK4/6i), CDK4/6i, and chemotherapy groups, respectively. Figure 3B shows OS in the expanded second-line treatment group (i.e. time from the start of initial CDK4/6i for AMBC); the 2-year OS rate was 83.3%, 89.8%, 92.9%, and 75.4% in the ET alone, molecularly-targeted therapy (excluding CDK4/6i), CDK4/6i, and chemotherapy groups, respectively.

**Fig. 4:**
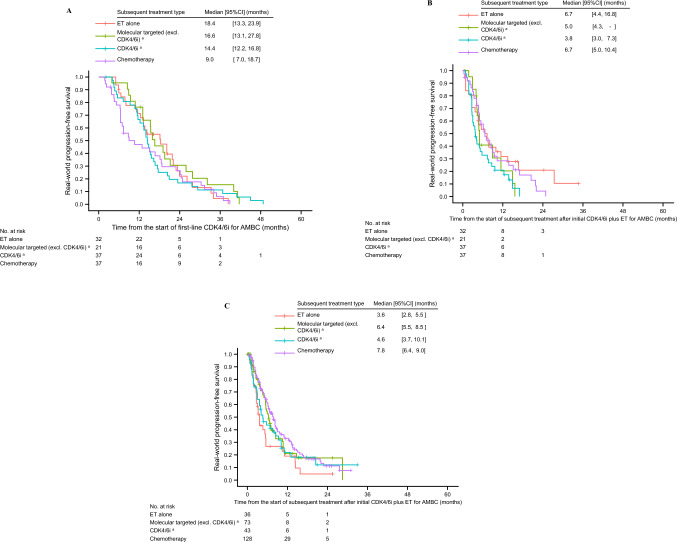
Real-world progression-free survival (rwPFS) in patients who received subsequent treatment after initial CDK4/6i plus ET by treatment type: rwPFS of initial CDK4/6i plus ET, defined as time from the start of first-line CDK4/6i therapy, in the exclusive first-line treatment group (**A**), rwPFS of subsequent treatment, defined as time from the start of subsequent treatment after initial CDK4/6i plus ET, in the exclusive first-line treatment group (**B**), and rwPFS of subsequent treatment, defined as time from the start of subsequent treatment after initial CDK4/6i plus ET, in the expanded second-line treatment group (**C**). *AMBC* advanced or metastatic breast cancer; *CDK4/6i* cyclin-dependent kinase 4/6 inhibitor; *CI* confidence interval; *ET* endocrine therapy. ^a^Treatment was provided with or without ET

Additionally, we analyzed rwPFS by subsequent treatment after initial CDK4/6i plus ET in order to clarify the efficacy of the subsequent treatment after initial CDK4/6i plus ET. Figure 4A shows rwPFS results for the initial CDK4/6i therapy (i.e. time from the start of first-line CDK4/6i) in the exclusive first-line treatment group patients, to determine whether the efficacy of initial CDK4/6i plus ET influenced subsequent treatment choices. In patients who received chemotherapy after the initial CDK4/6i plus ET, 1-year PFS rate was 46.9% (i.e. having disease progression within 1 year), whereas it was 71.2%, 76.2%, and 66.7% with ET alone, molecularly-targeted therapy, and CDK4/6i therapy, respectively. Figure 4B shows rwPFS results for the subsequent treatment after initial CDK4/6i plus ET (i.e. time from the start of subsequent treatment after CDK4/6i) in the exclusive first-line treatment group; median PFS showed a similar trend. We also analyzed rwPFS by subsequent treatment after initial CDK4/6i plus ET (i.e. time from the start of subsequent treatment after CDK4/6i) in the expanded second-line treatment group in order to investigate the effects of the post-CDK4/6i treatments in patients who had poor sensitivity to ET (Fig. 4C). Median rwPFS in patients who received ET alone was markedly shorter than in those who received molecularly-targeted therapy (ET alone [reference] vs molecularly-targeted therapy, log-rank *p* = 0.034) or chemotherapy (ET alone [reference] vs chemotherapy, log-rank *p* = 0.004).

## Discussion

The value of real-world data from routine clinical practice is increasingly recognized. While RCTs remain the gold standard, their findings may lack generalizability due to the strict selection criteria that do not always reflect general populations. The present study provides real-world evidence regarding the use in daily clinical practice of CDK4/6is currently available in Japan, namely palbociclib and abemaciclib. The findings may also help address the knowledge gap in the current evidence for treatment strategies after disease progression despite first- or second-line treatment with a CDK4/6i plus ET.

### Patient characteristics

In the present study, abemaciclib tended to be used in younger (premenopausal) patients, in both the first- and the second-line treatment groups (see Table 1). It is possible that the attending physician did not choose abemaciclib for older patients because of concerns about known adverse effects, such as diarrhea [23], a subjective symptom that may be difficult for elderly patients to self-manage. In an age-specific subgroup analysis of the MONARCH 2 and MONARCH 3 trials [24], moderately higher rates of various adverse events were reported in older patients receiving abemaciclib plus ET vs placebo plus ET; however, these outcomes were manageable by dose adjustments and concomitant medication. In terms of efficacy, median PFS did not differ among age subgroups, indicating that abemaciclib plus ET provides consistent benefits regardless of age.

We hypothesized that employed patients might be concerned about experiencing diarrhea while at work, making them less likely to choose abemaciclib, which has a higher incidence of diarrhea as an adverse event. In the present study, no differences were observed in this aspect between patients receiving palbociclib versus those receiving abemaciclib (see Table 1), indicating that patients’ employment status at baseline did not affect the choice of CDK4/6i.

In the first-line treatment group, patients with TFI < 12 months, high Ki67, high NOLUS, liver metastasis, and history of perioperative chemotherapy were more likely to have received abemaciclib (see Table 1). In other words, abemaciclib tended to be chosen in cases in which the prognosis was considered poor. The precise reasons for this are unclear. However, an exploratory pooled analysis of the MONARCH 2 and MONARCH 3 trials showed that while abemaciclib provided consistent benefits across all subgroups, its effect was greatest in patients with poor prognostic factors (e.g. presence of liver metastasis) [25]. The findings may partly influence physicians’ decision to choose a CDK4/6i for the treatment of such patients.

### Survival outcomes by treatment line

The survival outcomes of the present study for patients with de novo stage IV disease and TFI ≥ 12 months in the first-line treatment group (see Fig. 2A) are in line with those reported in the phase 3 RCTs, namely, MONARCH 3 (median PFS, 28.18 months with abemaciclib plus non-steroidal AI) [11] and MONALEESA 2 (median PFS, 25.3 months with ribociclib plus non-steroidal AI) [26]. These patients are considered sensitive to ET, and a good response to the first-line CDK4/6i therapy was confirmed. The findings are also consistent with those of a previous study of real-world clinical outcomes of palbociclib plus ET in Japan [27]; median rwPFS was 33.6, 14.5, and 27.3 months for patients in the first-line treatment group with de novo metastatic disease, TFI < 12 months, and TFI ≥ 12 months, respectively.

By contrast, in the present study, patients with TFI < 12 months had shorter median rwPFS than those with de novo stage IV disease or TFI ≥ 12 months (see Fig. 2A). This finding can be interpreted as suggesting that patients with TFI < 12 months may have a tumor resistant to ET. The benefit of CDK4/6i is reported to be limited in patients who had tumors with intrinsic endocrine resistance [28]. Therefore, even when the same initial regimen (i.e. CDK4/6i plus ET) was given, the outcomes largely differ due to a lack of efficacy of ET.

Similarly to rwPFS, median CFS in the first-line treatment group was shorter in patients with TFI < 12 months than in those with de novo stage IV disease or TFI ≥ 12 months (see Fig. 2B). These outcomes align with the findings from MONARCH 3 (median CFS, 46.7 months) [14]. Furthermore, in the second-line treatment group, median CFS (i.e. time from the start of the second-line treatment) was 24.8 months, which is consistent with the results of MONARCH 2 (median CFS 24.56 months with second-line abemaciclib plus fulvestrant) [8], as well as the above-mentioned real-world clinical data from Japan (median CFS 23.1 months with second-line palbociclib plus ET) [27]. Together, our real-world data reflect those of RCTs as well as real-world evidence from Japan available to date, supporting their reliability.

We also found that the survival outcomes were similar regardless of CDK4/6i used (see Supplementary Fig. 1- 3). However, no conclusions can be drawn from this observation, because no direct comparison was made, abemaciclib was more often used to treat patients whose prognosis was considered poor, and patients’ background factors vary.

### Type of endocrine therapy used with CDK4/6i

Because patients who have relapse within 12 months of completing adjuvant ET are deemed to have resistance to ET [29], which would confer a poor response to CDK4/6i therapy, we analyzed data from these patients separately so as to assess the true effect of first-line CDK4/6i plus ET in endocrine-sensitive patients; thus, patients were classified as the exclusive first-line treatment group (i.e. those with de novo stage IV disease or TFI ≥ 12 months) and the expanded second-line treatment group (i.e. those with TFI < 12 months and those in the second-line treatment group).

In the exclusive first-line treatment group, there was no difference in rwPFS by type of ET (fulvestrant or AI) used in combination with CDK4/6i therapy (see Supplementary Fig. 4). Therefore, in endocrine-sensitive patients who are likely to respond well to CDK4/6i plus ET, either fulvestrant or AI can be a partner of CDK4/6i as first-line treatment for AMBC. However, *ESR1* mutations, a known cause of acquired resistance to AI in the treatment of HR-positive AMBC, may arise [30], potentially affecting long-term prognosis. Therefore, the impact of *ESR1* mutations on survival, especially OS, should carefully be examined.

### Type and outcomes of subsequent treatment after initial CDK4/6i plus ET

Of the patients who had their treatment switched to chemotherapy due to disease progression during initial CDK4/6i plus ET, early deaths were observed in both the exclusive first-line and the expanded second-line treatment groups (see Fig. 3A and B, respectively). The fact that chemotherapy was required immediately after CDK4/6i plus ET may indicate that they have poor sensitivity to ET and prognosis is expected to be poor (see Fig. 4A). Among chemotherapeutic agents, oral fluoropyrimidine carbamates (S-1 and capecitabine) were most frequently used. S-1 has been shown to be non-inferior to taxanes in terms of OS as first-line chemotherapy for HER2-negative metastatic breast cancer [31]; however, its effects on PFS and objective response rate are less certain. In the recently reported results of the RESQ study, which compared eribulin vs S-1 as first- or second-line chemotherapy for HER2-negative metastatic breast cancer, median OS was significantly longer with eribulin [32]. Therefore, the choice of chemotherapeutic agents after CDK4/6i plus ET should be carefully considered, especially for patients with poor prognostic factors. New treatment strategies, including use of a next-generation antibody–drug conjugate (e.g. trastuzumab deruxtecan), may be considered for these patients.

Another finding of the analysis of post-CDK4/6i treatment is that in the exclusive first-line treatment group, median rwPFS of the subsequent treatment after CDK4/6i plus ET did not reach 7 months, regardless of the type of subsequent treatment (see Fig. 4B). The results were similar to those for ET alone in the RCTs of post-CDK4/6i treatment: median PFS with fulvestrant alone (the control) was 5.3 months in the postMONARCH trial [17, 33] and 3.6 months in the CAPItello-291 trial [34]. Therefore, although only a small number of patients who had disease progression and received subsequent treatment were included in the present study, outcomes of post-CDK4/6i treatment remain insufficient, highlighting the need for individualized treatment approach that can overcome the resistance mechanism.

In the expanded second-line treatment group, median rwPFS of subsequent treatment after CDK4/6i therapy was shorter with ET alone and with continuous CDK4/6i therapy (see Fig. 4C). Moreover, rwPFS was markedly shorter with ET alone than with molecularly-targeted therapy or chemotherapy (see Fig. 4C). These findings suggest that not only ET alone but also continuous CDK4/6i therapy may not be a suitable option in such patients, who are unlikely to respond to ET.

Further identification of the characteristics of patients most likely to be ET-resistant may be necessary. In the analysis of the association of intrinsic subtypes with PFS in HR-positive HER2-negative AMBC patients included in the MONALEESA trials [35], basal-like subtype was associated with poor outcome and lack of benefit from CDK4/6i (ribociclib), whereas HER2-enriched subtype showed the worst prognosis with ET alone but the greatest relative benefit with ribociclib and ET. Although intrinsic subtypes were not determined in the present study, patients who progressed within 1 year after CDK4/6i therapy may be those with poor prognostic factors, who would therefore benefit from other treatment strategies.

### Limitations

This observational study, which analyzed real-world data, has several limitations. Because data were collected from existing data source, some variables were missing or insufficient. For some patients, performance status was missing in the medical record and therefore judged retrospectively (as per the procedure described in the footnote of Table 1). Disease progression was determined based on data in the medical records of each patient, so it was judged by their attending physicians, and CT scan intervals varied. Analyses of survival outcomes by post-CDK4/6i therapy were not adjusted for cofounding factors, because of the small number of patients who had early disease progression and required subsequent treatment.

Investigation of the incidence of adverse events is a standard aspect of real-world studies. However, the present study aimed to identify the characteristics of patients who had received a CDK4/6i plus ET, and to analyze their survival outcomes. Additionally, we expected the physicians to have based their choice of CDK4/6i on the results of previous studies. Furthermore, we believe there was little possibility of finding more valuable evidence from this study than what had already been obtained from the numerous large clinical trials previously reported. For these reasons, detailed adverse event data were not collected, although we recorded reasons for discontinuing initial CDK4/6i plus ET (e.g. progression of disease, adverse events, financial reasons).

## Conclusions

This is the first large-scale observational study that provides real-world evidence regarding palbociclib and abemaciclib (CDK4/6is currently available in Japan) plus ET as first- or second-line treatment for HR-positive, HER2-negative AMBC in Japan. Bearing in mind its limitations, and given that prospective RCT data are especially lacking for post-CDK4/6i therapy options, we hope that our real-world analysis data will help physicians in the clinical decision-making process. Consistent with previous phase 3 RCTs, favorable outcomes of the first- and second-line CDK4/6i plus ET were confirmed; however, outcomes of subsequent treatment after initial CDK4/6i plus ET were insufficient, especially for those who had disease progression within 1 year. New treatment strategies that are tailored to an individual patient are necessary.

## Supplementary Information

Below is the link to the electronic supplementary material.

Supplementary file1 (PPTX 135 KB)

## Data Availability

The datasets are not publicly available due to privacy/ethical restrictions but are available from the corresponding author on reasonable request.
