# Correction: Real‑world evidence from Japan regarding survival outcomes and treatment sequence in patients receiving CDK4/6 inhibitor plus endocrine therapy as first‑ or second‑line treatment for hormone receptor–positive, HER2‑negative advanced or metastatic breast cancer

**DOI:** 10.1007/s12282-025-01736-0

**Published:** 2025-06-23

**Authors:** Tetsuhiro Yoshinami, Yuko Takano, Yukinori Ozaki, Yukiko Kajiwara, Mitsugu Yamamoto, Ken-ichi Watanabe, Masami Tsukabe, Fumie Fujisawa, Shigenori E. Nagai, Nobuhiro Shibata, Chiya Oshiro, Hiroko Bando, Nobuyuki Tsunoda, Kazuhiko Yamagami, Kei Koizumi, Masahiro Takada, Naoko Toriguchi, Nobuyuki Sekine, Tsutomu Kawaguchi, Shigehira Saji, Yasuaki Sagara, Satoshi Morita, Norikazu Masuda

**Affiliations:** 1 Department of Breast and Endocrine Surgery, Graduate School of Medicine, Osaka University, 2-2-E10 Yamadaoka, Suita, Osaka 565-0871 Japan; 2 Department of Breast and Endocrine Surgery, Nagoya University Hospital, Nagoya, Aichi Japan; 3 Department of Breast Medical Oncology, The Cancer Institute Hospital of JFCR, Tokyo, Japan; 4 Department of Breast Surgery, Hiroshima City Hiroshima Citizens Hospital, Hiroshima, Japan; 5 Department of Breast Oncology, National Hospital Organization Hokkaido Cancer Center, Sapporo, Hokkaido Japan; 6 Department of Medical Oncology, Shiga General Hospital, Moriyama, Shiga Japan; 7 Division of Breast Oncology, Saitama Cancer Center, Kitaadachi-gun, Saitama, Japan; 8 Department of Clinical Oncology, Kansai Medical University Hospital, Hirakata, Osaka Japan; 9 Department of Breast Surgery, Kaizuka City Hospital, Kaizuka, Osaka Japan; 10 Department of Breast and Endocrine Surgery, Institute of Medicine, University of Tsukuba, Tsukuba, Ibaraki Japan; 11 Department of Breast Surgery, Japanese Red Cross Aichi Medical Center Nagoya Daiichi Hospital, Nagoya, Aichi Japan; 12 Department of Breast Surgery and Oncology, Shinko Hospital, Kobe, Hyogo Japan; 13 Department of Surgery 1, Division of Breast Surgery, Hamamatsu University School of Medicine, Hamamatsu, Shizuoka Japan; 14 Department of Breast Surgery, Kansai Medical University, Hirakata, Osaka Japan; 15 Department of Japan Drug Development and Medical Affairs, Eli Lilly Japan K.K, Kobe, Hyogo Japan; 16 Department of Medical Oncology, Fukushima Medical University, Fukushima, Japan; 17 Department of Breast and Thyroid Surgical Oncology, Social Medical Corporation Hakuaikai Sagara Hospital, Kagoshima, Japan; 18 Department of Biomedical Statistics and Bioinformatics, Graduate School of Medicine, Kyoto University, Kyoto, Japan; 19 Department of Breast Surgery, Graduate School of Medicine, Kyoto University, Kyoto, Japan

**Correction: Breast Cancer (2025)** 10.1007/s12282-025-01713-7

In the sentence beginning ‘Anonymized data were collected …’ in this article, the text ‘40 participating institutions’ should have read ‘34 participating institutions’.

