# Supplementary material for: Real-world evidence from Japan regarding survival outcomes and treatment sequence in patients receiving CDK4/6 inhibitor plus endocrine therapy as first- or second-line treatment for hormone receptor–positive, HER2-negative advanced or metastatic breast cancer
Source: Breast Cancer. 2025 May 20;32(4):841–56. doi: 10.1007/s12282-025-01713-7 (PMC12174191; doi:10.1007/s12282-025-01713-7)
Supplement: Supplementary file 1 — Supplementary file1 (PPTX 135 KB) [file 12282_2025_1713_MOESM1_ESM.pptx]

## Slide 1
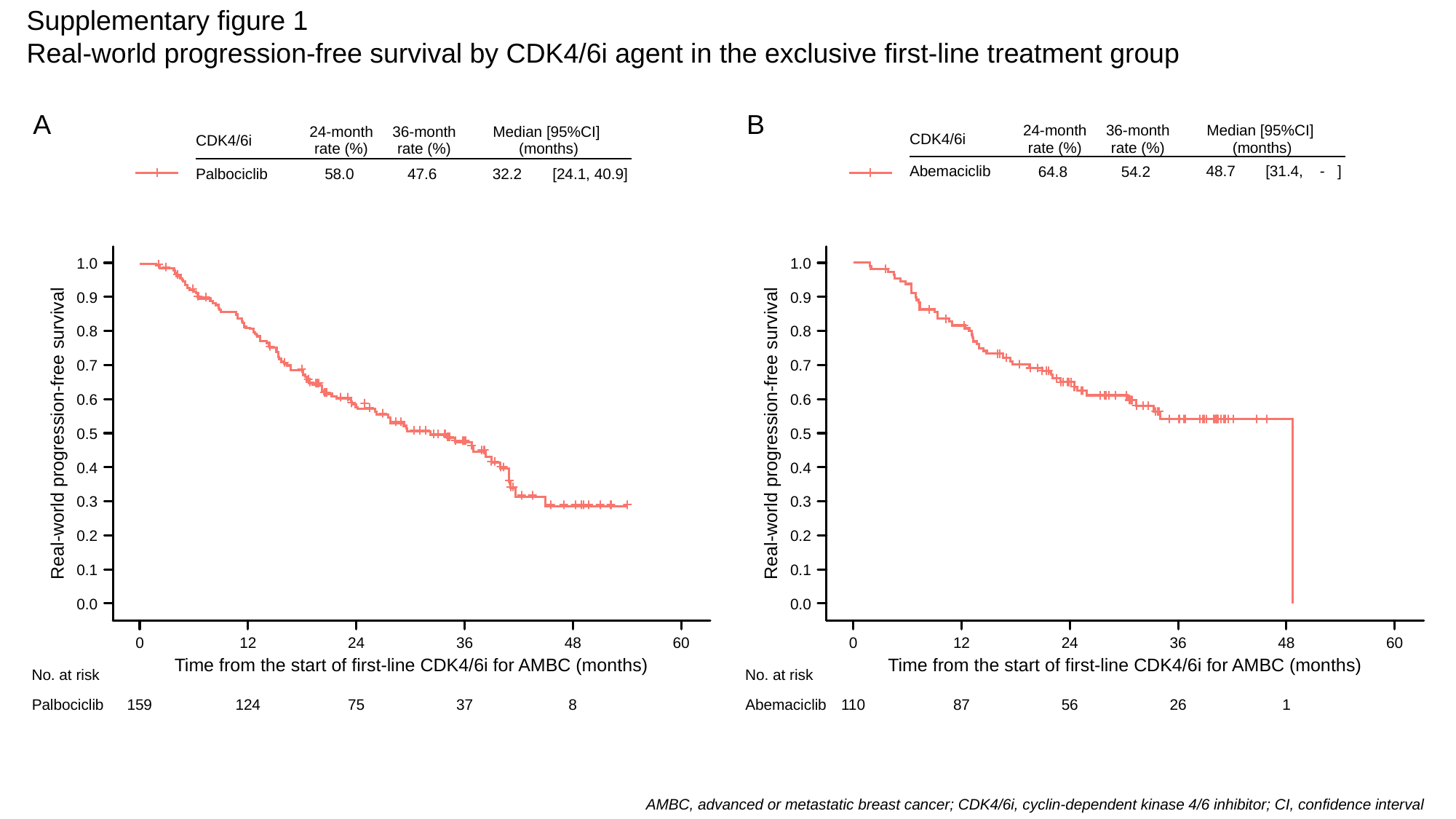

Supplementary figure 1
Real-world progression-free survival by CDK4/6i agent in the exclusive first-line treatment group
A
B
| CDK4/6i | 24-month rate (%) | 36-month rate (%) | Median [95%CI] (months) | |
| --- | --- | --- | --- | --- |
| Palbociclib | 58.0 | 47.6 | 32.2 | [24.1, 40.9] |
| CDK4/6i | 24-month rate (%) | 36-month rate (%) | Median [95%CI] (months) | |
| --- | --- | --- | --- | --- |
| Abemaciclib | 64.8 | 54.2 | 48.7 | [31.4, - ] |
1.0
1.0
Real-world progression-free survival
Real-world progression-free survival
0.9
0.9
0.8
0.8
0.7
0.7
0.6
0.6
0.5
0.5
0.4
0.4
0.3
0.3
0.2
0.2
0.1
0.1
0.0
0.0
0
12
24
36
48
60
0
12
24
36
48
60
Time from the start of first-line CDK4/6i for AMBC (months)
Time from the start of first-line CDK4/6i for AMBC (months)
No. at risk
No. at risk
Palbociclib
159
124
75
37
8
Abemaciclib
110
87
56
26
1
AMBC, advanced or metastatic breast cancer; CDK4/6i, cyclin-dependent kinase 4/6 inhibitor; CI, confidence interval

## Slide 2
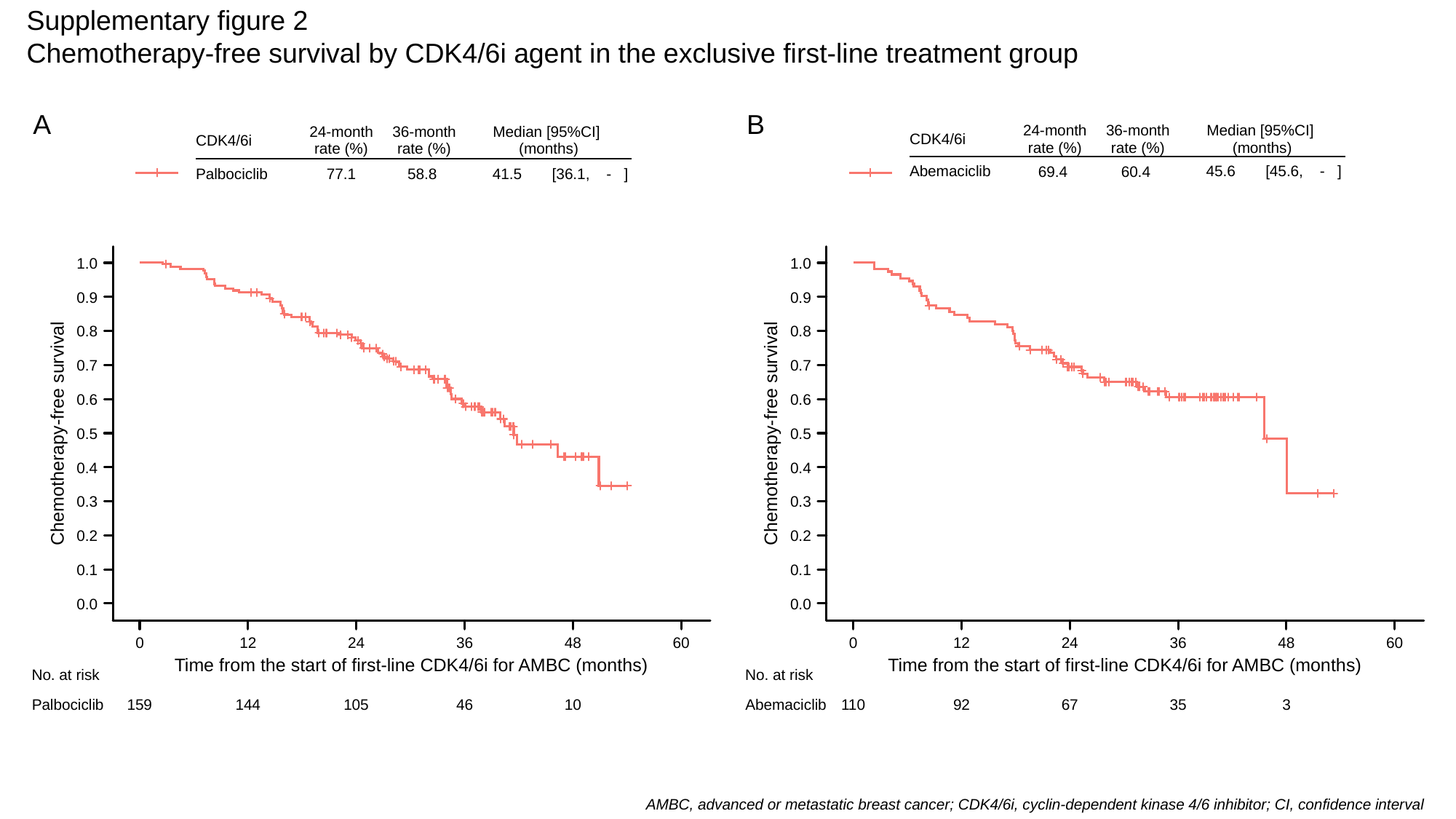

Supplementary figure 2
Chemotherapy-free survival by CDK4/6i agent in the exclusive first-line treatment group
A
B
| CDK4/6i | 24-month rate (%) | 36-month rate (%) | Median [95%CI] (months) | |
| --- | --- | --- | --- | --- |
| Palbociclib | 77.1 | 58.8 | 41.5 | [36.1, - ] |
| CDK4/6i | 24-month rate (%) | 36-month rate (%) | Median [95%CI] (months) | |
| --- | --- | --- | --- | --- |
| Abemaciclib | 69.4 | 60.4 | 45.6 | [45.6, - ] |
1.0
1.0
0.9
0.9
Chemotherapy-free survival
Chemotherapy-free survival
0.8
0.8
0.7
0.7
0.6
0.6
0.5
0.5
0.4
0.4
0.3
0.3
0.2
0.2
0.1
0.1
0.0
0.0
0
12
24
36
48
60
0
12
24
36
48
60
Time from the start of first-line CDK4/6i for AMBC (months)
Time from the start of first-line CDK4/6i for AMBC (months)
No. at risk
No. at risk
Palbociclib
159
144
105
46
10
Abemaciclib
110
92
67
35
3
AMBC, advanced or metastatic breast cancer; CDK4/6i, cyclin-dependent kinase 4/6 inhibitor; CI, confidence interval

## Slide 3
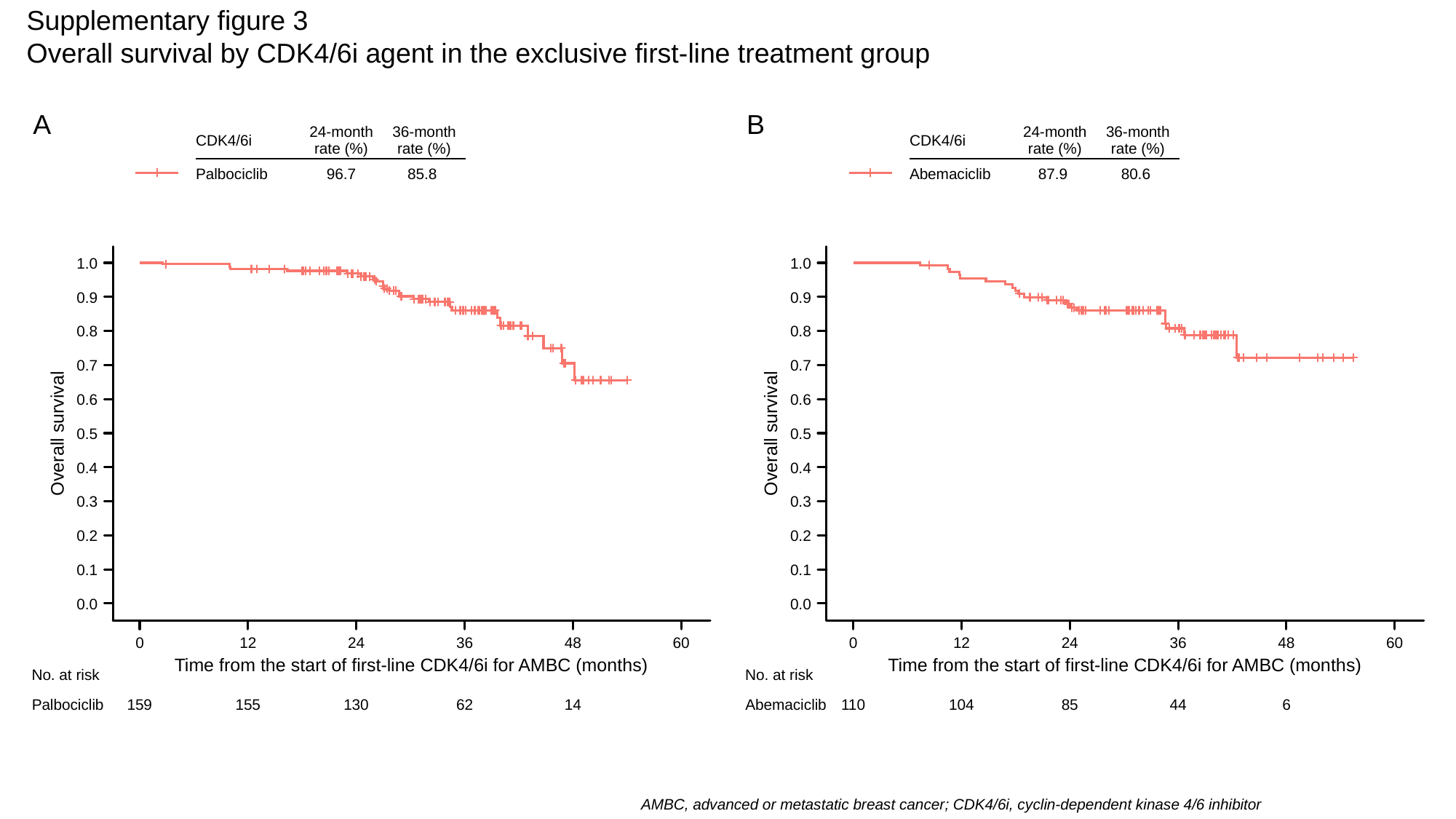

Supplementary figure 3
Overall survival by CDK4/6i agent in the exclusive first-line treatment group
A
B
| CDK4/6i | 24-month rate (%) | 36-month rate (%) |
| --- | --- | --- |
| Palbociclib | 96.7 | 85.8 |
| CDK4/6i | 24-month rate (%) | 36-month rate (%) |
| --- | --- | --- |
| Abemaciclib | 87.9 | 80.6 |
1.0
1.0
0.9
0.9
0.8
0.8
0.7
0.7
Overall survival
Overall survival
0.6
0.6
0.5
0.5
0.4
0.4
0.3
0.3
0.2
0.2
0.1
0.1
0.0
0.0
0
12
24
36
48
60
0
12
24
36
48
60
Time from the start of first-line CDK4/6i for AMBC (months)
Time from the start of first-line CDK4/6i for AMBC (months)
No. at risk
No. at risk
Palbociclib
159
155
130
62
14
Abemaciclib
110
104
85
44
6
AMBC, advanced or metastatic breast cancer; CDK4/6i, cyclin-dependent kinase 4/6 inhibitor

## Slide 4
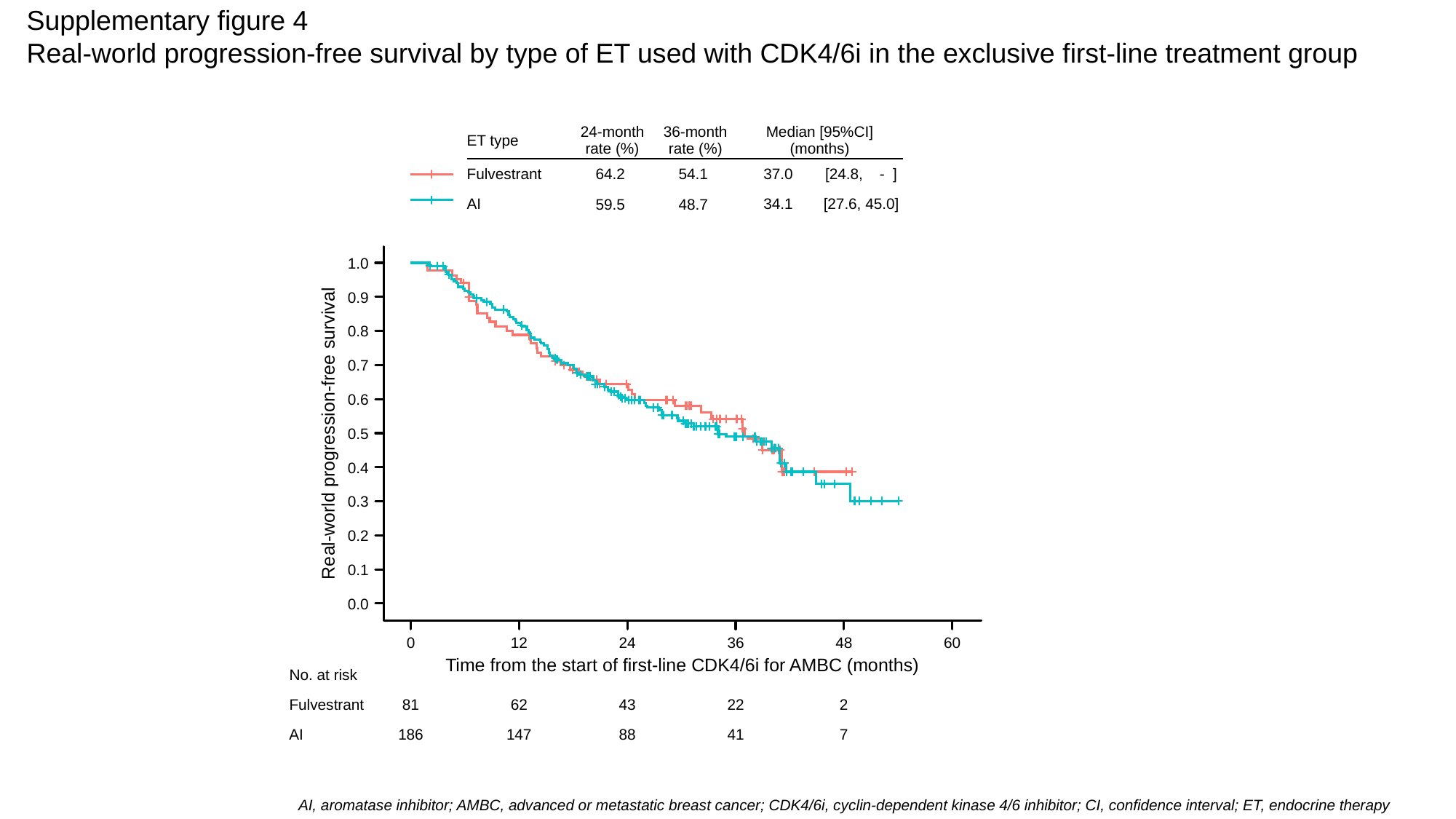

Supplementary figure 4
Real-world progression-free survival by type of ET used with CDK4/6i in the exclusive first-line treatment group
| ET type | 24-month rate (%) | 36-month rate (%) | Median [95%CI] (months) | |
| --- | --- | --- | --- | --- |
| Fulvestrant | 64.2 | 54.1 | 37.0 | [24.8, - ] |
| AI | 59.5 | 48.7 | 34.1 | [27.6, 45.0] |
1.0
Real-world progression-free survival
0.9
0.8
0.7
0.6
0.5
0.4
0.3
0.2
0.1
0.0
0
12
24
36
48
60
Time from the start of first-line CDK4/6i for AMBC (months)
No. at risk
Fulvestrant
81
62
43
22
2
AI
186
147
88
41
7
AI, aromatase inhibitor; AMBC, advanced or metastatic breast cancer; CDK4/6i, cyclin-dependent kinase 4/6 inhibitor; CI, confidence interval; ET, endocrine therapy
